# Valorization of CBD-hemp through distillation to provide essential oil and improved cannabinoids profile

**DOI:** 10.1038/s41598-021-99335-4

**Published:** 2021-10-06

**Authors:** Valtcho D. Zheljazkov, Filippo Maggi

**Affiliations:** 1 Crop and Soil Science Department, Oregon State University, 3050 SW Campus Way, Corvallis, OR 97331 USA; 2 School of Pharmacy, University of Camerino, via Sant’ Agostino 1, 62032 Camerino, Italy

**Keywords:** Chemical biology, Plant sciences, Chemistry

## Abstract

Hemp (*Cannabis sativa* L.) synthesizes and accumulates a number of secondary metabolites such as terpenes and cannabinoids. They are mostly deposited as resin into the glandular trichomes occurring on the leaves and, to a major extent, on the flower bracts. In the last few years, hemp for production of high-value chemicals became a major commodity in the U.S. and across the world. The hypothesis was that hemp biomass valorization can be achieved through distillation and procurement of two high-value products: the essential oil (EO) and cannabinoids. Furthermore, the secondary hypothesis was that the distillation process will decarboxylate cannabinoids hence improving cannabinoid composition of extracted hemp biomass. Therefore, this study elucidated the effect of steam distillation on changes in the content and compositional profile of cannabinoids in the extracted biomass. Certified organic CBD-hemp strains (chemovars, varieties) Red Bordeaux, Cherry Wine and Umpqua (flowers and some upper leaves) and a T&H strain that included chopped whole-plant biomass, were subjected to steam distillation, and the EO and cannabinoids profile were analyzed by gas chromatography-mass spectrometry (GC–MS) and HPLC, respectively. The distillation of hemp resulted in apparent decarboxylation and conversion of cannabinoids in the distilled biomass. The study demonstrated a simple method for valorization of CBD-hemp through the production of two high-value chemicals, i.e. EO and cannabinoids with improved profile through the conversion of cannabidiolic acid (CBD-A) into cannabidiol (CBD), cannabichromenic acid (CBC-A) into cannabichromene (CBC), cannabidivarinic acid (CBDV-A) into cannabidivarin (CBDV), cannabigerolic acid (CBG-A) into cannabigerol (CBG), and δ-9-tetrahydrocannabinolic acid (THC-A) into δ-9-tetrahydrocannabinol (THC). In addition, the distilled biomass contained CBN while the non-distilled did not. Distillation improved the cannabinoids profile; e.g. the distilled hemp biomass had 3.4 times higher CBD in variety Red Bordeaux, 5.6 times in Cherry Wine, 9 times in variety Umpqua, and 6 times in T&H compared to the original non-distilled samples, respectively. Most of the cannabinoids remained in the distilled biomass and small amounts of CBD were transferred to the EO. The CBD concentration in the EO was as follows: 5.3% in the EO of Umpqua, 0.15% in the EO of Cherry Wine and Red Bordeaux and 0.06% in the EO of T&H. The main 3 EO constituents were similar but in different ratio; myrcene (23.2%), (*E*)-caryophyllene (16.7%) and selina-3,7(11)-diene (9.6%) in Cherry Wine; (*E*)-caryophyllene (~ 20%), myrcene (16.6%), selina-3,7(11)-diene (9.6%), α-humulene (8.0%) in Red Bordeaux; (*E*)-caryophyllene (18.2%) guaiol (7.0%), 10-*epi*-γ-eudesmol (6.9%) in Umpqua; and (*E*)-caryophyllene (30.5%), α-humulene (9.1%), and (*E*)-α-bisabolene (6.5%) in T&H. In addition, distillation reduced total THC in the distilled biomass. Scanning electron microscopy (SEM) analyses revealed that most of the glandular trichomes in the distilled biomass were not disturbed (remained intact); that suggest a possibility for terpenes evaporation through the epidermal membrane covering the glandular trichomes leaving the cannabinoids in the trichomes. This explained the fact that distillation resulted in terpene extraction while the cannabinoids remained in the distilled material.

## Introduction

Industrial hemp (*Cannabis sativa* L.) was grown as a commodity fiber crop in North America until the mid-1930s. Hemp was banned and was considered an illegal crop in the United States for several decades. In 2014, section 7606 of the U.S. Congress Agricultural Act of 2014, the “Farm Bill”, authorized pilot programs on cultivation of industrial hemp, defined as “the plant *Cannabis sativa* L. and any part of such plant, whether growing or not, with a delta-9 tetrahydrocannabinol (THC) concentration of not more than 0.3% on a dry weight basis”. The 2018 Farm Bill decriminalized cultivation of industrial hemp and designated the U.S. Department of Agriculture (USDA) Agricultural Marketing Service to develop regulations. Hemp production in the U.S. is increasing rapidly and there were up to 500,000 licensed acres to grow hemp in 2019^1^, that would have produced $11.3 billion of income, or around 6% of the total value of all cash crops in this country^1^. Currently, at least 47 states have passed legislation to establish hemp production programs or allow for hemp cultivation research. At this time, hemp is prohibited only in Idaho, and Mississippi. Specific state legislation varies from state to state. Currently, Oregon legal environment with respect to commercial hemp production is among the most reassuring in the U.S. and hence, stimulating hemp production for high-value chemicals.

Most of the hemp grown in the U.S. is for production of high-value chemicals such as cannabinoids and terpenes. Essential oil (EO) production is a novel use of hemp, and as such, it needs to be researched. Hemp for EO and cannabinoids production is an understudied, high-value crop, with many pending unanswered questions.

Hemp synthesizes and accumulates numerous secondary metabolites^2–4^. The most important of these are the cannabinoids and terpenes; they are toxic to many organisms and are considered to be plant protective chemicals. Hemp chemicals have numerous uses due to their bioactivities^5–10^.

Hemp (*C. sativa*) is an annual, normally wind pollinated dioecious plant (separate male and female plants), although monoecious forms can also occur naturally. Botanically, hemp belongs to Cannabaceae. There has been a debate on whether hemp is a single species or include other species such as *Cannabis indica* Lam. and *Cannabis ruderalis* Janisch. Small and Cronquist^11^ separated the species into two subspecies, subsp. *indica* (Lam.) E. Small & Cronq., with relatively high amounts of the psychotropic constituent THC, and subsp. *sativa* with low amounts of THC. According to this systematics, the modern fiber and grain industrial hemp varieties would belong to subsp. *sativa*. Therefore, most recreational, or medical marijuana varieties and strains would belong to subsp. *indica*. However, there are numerous hybrids blurring the line. Overall, botanists consider *C. sativa* to be a single species with several subspecies^12–14^.

**Figure 1:**
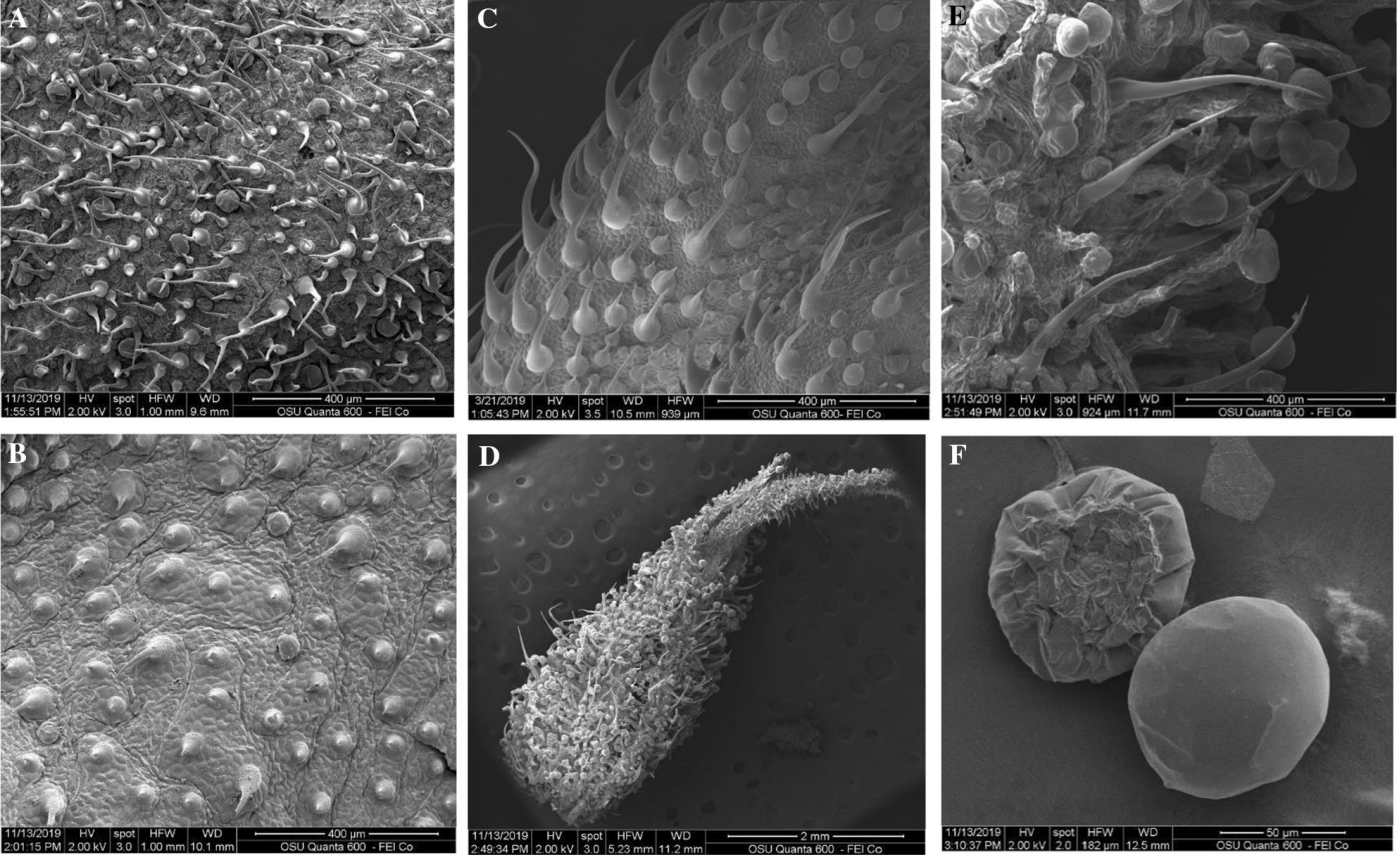
(**A**) Hemp abaxial (lower) leaf surface with glandular trichomes, and slender cystolithic non glandular trichomes. (**B**) Hemp adaxial (upper) leaf surface with an abundance of cystolithic trichomes and few sessile glandular trichomes. (**C**) Hemp leaf petiole with an abundance of cystolithic and slender non glandular trichomes and few sessile glandular trichomes. (**D**) Flower bract densely covered with glandular trichomes. (**E**) Close up of flower bract with glandular trichomes and slender non glandular trichomes. (**F**) Detached sessile glandular trichomes from hemp leaves.

**Figure 2:**
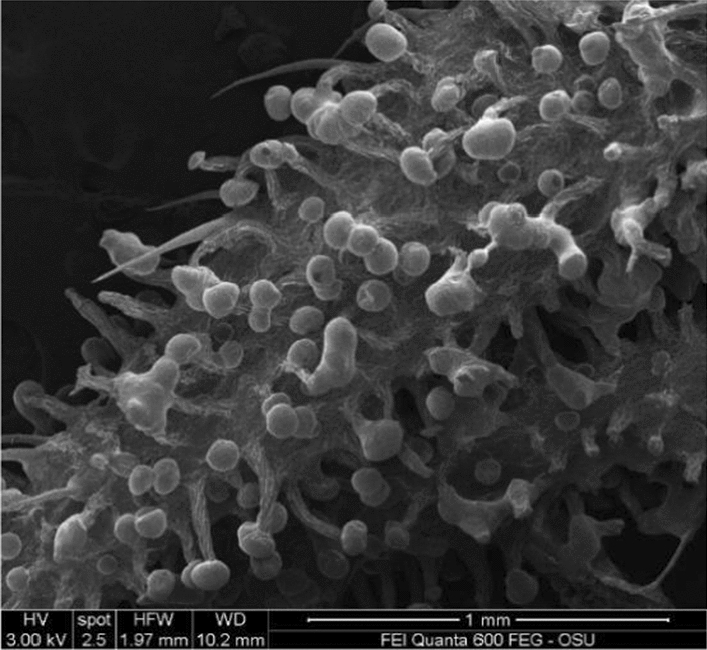
Non-extracted Red Bordeaux flower part with glandular trichomes.

Hemp plants form different epidermal trichomes, which are considered defense structures to reduce herbivory by making the biomass less palatable. Cystolith trichomes contain calcium carbonate particles. These trichomes are present in great numbers on both leaf surfaces along with the slender non glandular trichomes^13^. In addition, hemp forms secretory or glandular trichomes, the sites for EO (terpenes) synthesis and accumulation, with the highest density in non-fertilized flower bracts (Figs. 1, 2). Current understanding is that secretory trichomes are also the site where cannabinoids are synthesized and accumulate^3,14,15^. Most of the hemp chemicals are produced in multicellular glandular trichomes, which can be sessile glands (with very short stalks), or long-stalk secretory glands (Figs. 1, 2). The top of these glands is a cavity covered by a waxy cuticle, where the resin (a mix of cannabinoids and terpenes) is accumulated. Since the waxy cuticle of the glands is a thin layer, it can easily be ruptured resulting in a release of its contents. The density of secretory glands differs, with the highest concentration found in perigonal bracts covering the female flowers. Therefore, traditionally, flowers have been the plant part of the most interest because of their high content of various natural products^2,14,15^.

Hemp plants contain a whole array of chemicals that may act synergistically or antagonistically. Currently, the pharmacological power of the *C. sativa* is based on the content of δ-9-tetrahydrocannabinolic acid (THC-A) and cannabidiolic acid (CBD-A)^16^. Other major cannabinoids include cannabinolic acid (CBN-A), cannabigerolic acid (CBG-A), cannabichromenic acid (CBC-A), and cannabinodiolic acid (CBND-A)^2,17^. With recent legalization of hemp in many countries, researchers are now focusing on better understanding of the role of various other chemicals found in hemp^2,18^. Terpenes (that are constituents of the hemp EO) contribute to the aroma of various hemp genotypes, and so far, around 140 different terpenes have been reported in hemp^2,14,19,20^. The major ones belong to the class of monoterpenes (e.g., α-pinene and myrcene) and sesquiterpenes ((*E*)-caryophyllene, and  caryophyllene oxide)^21^.

The hypothesis was that CBD-hemp biomass valorization can be achieved through distillation and production of two high-value products: EO and cannabinoids. Furthermore, a preliminary distillation process may decarboxylate cannabinoids and therefore improve cannabinoid composition of extracts from the residual biomass.

## Results

### Essential oil (EO) content (yield) and composition of Cherry Wine (CW), Red Bordeaux (RB), Umpqua (Umpq) and T&H

The EO yield (% in dry biomass) was highest in CW and RB (1.85 and 1.6%, respectively), lower in Umpqua (0.72%), and the lowest in T&H (0.37%) strains (Table 1). The lower EO content in T&H was most probably because the biomass was chopped by the grower; it included all plant parts (stems, leaves, flowers), and therefore there is dilution factor in addition to the chopping that may have destroyed some of the glandular trichomes resulting in terpene evaporation.

**Table 1:** Essential oil yield and composition obtained by non-stop steam distillation for 240 min of autoflower type hemp biomass of Cherry Wine organic (CW), Red Bordeaux organic (RB), Umpqua organic (Umpq), and non-stop steam distillation for 120 min of chopped biomass of autoflower type hemp T&H.

Essential oil yield and composition	RT (min)^a^	RI exp.^b^	RI lit.	Variety			
				CW	RB	Umpq	T&H chopped
				%			
Essential oil yield	–			1.85 ± 0.06	1.60 ± 0.05	0.72 ± 0.04	0.37 ± 0.03
5,5-Dimethyl-1-vinylbicyclo[2.1.1]hexane	3.48	914	920	0.02 ± 0.01	0.03 ± 0.00	0.00 ± 0.00	0.07 ± 0.00
α-Thujene	3.62	920	924	0.01 ± 0.00	0.01 ± 0.00	–	–
α-Pinene	3.73	925	932	0.42 ± 0.00	1.73 ± 0.40	2.68 ± 1.21	2.29 ± 1.26
Camphene	4.02	939	946	0.06 ± 0.00	0.07 ± 0.01	0.08 ± 0.04	0.04 ± 0.00
Sabinene	4.61	965	969	0.01 ± 0.00	0.01 ± 0.00	–	–
β-Pinene	4.65	967	974	0.89 ± 0.01	1.35 ± 0.16	1.60 ± 0.53	1.06 ± 0.52
Myrcene	5.13	989	988	23.15 ± 1.01	16.63 ± 1.05	3.38 ± 0.87	4.4 ± 1.88
*p*-cymene	6.11	1021	1020	0.02 ± 0.00	0.02 ± 0.01	–	–
Limonene	6.21	1024	1024	5.43 ± 0.32	4.85 ± 0.03	4.80 ± 0.87	0.82 ± 0.32
1,8-Cineole	6.26	1026	1026	0.15 ± 0.04	0.17 ± 0.01	–	–
(*Z*)-β-ocimene	6.64	1037	1032	0.03 ± 0.01	0.01 ± 0.00	–	–
(*E*)-β-ocimene	6.95	1046	1044	2.57 ± 0.22	0.78 ± 0.01	–	–
γ-Terpinene	7.26	1055	1054	0.01 ± 0.00	0.01 ± 0.00	–	–
Terpinolene	8.26	1084	1086	–	–	0.04 ± 0.00	–
Linalool	8.77	1100	1095	–	–	0.84 ± 0.13	–
*endo*-Fenchol	9.08	1108	1114	0.14 ± 0.03	0.12 ± 0.01	0.35 ± 0.05	0.03 ± 0.00
(*E*)-pinene hydrate	9.35	1116	1119	0.07 ± 0.01	0.07 ± 0.01	0.15 ± 0.03	0.01 ± 0.00
Borneol	10.96	1160	1165	0.01 ± 0.00	0.01 ± 0.00	0.03 ± 0.00	–
α-Terpineol	11.95	1188	1186	–	–	0.04 ± 0.01	–
α-Ylangene	17.95	1361	1373	–	0.02 ± 0.00	–	–
Sativene	18.57	1380	1390	–	–	0.02 ± 0.00	0.01 ± 0.00
(*Z*)-caryophyllene	19.05	1395	1408	0.01 ± 0.00	0.03 ± 0.00	–	0.27 ± 0.02
Sesquithujene	19.25	1400	1405	–	–	0.00 ± 0.00	0.01 ± 0.00
(*E*)-caryophyllene	19.45	1407	1417	16.72 ± 1.64	19.98 ± 0.52	18.20 ± 0.15	30.47 ± 0.27
(*Z*)-α-bergamotene	19.57	1412	1411	–	–	–	0.01 ± 0.00
α-Santalene	19.58	1411	1416	–	–	–	0.02 ± 0.00
γ-Elemene	19.99	1425	1434	1.36 ± 0.32	1.68 ± 0.09	0.00 ± 0.00	0.58 ± 0.01
*(E)*-α-bergamotene	20.11	1428	1432	1.55 ± 0.20	1.55 ± 0.00	2.13 ± 0.01	5.07 ± 0.05
α-Guaiene	20.12	1430	1437	–	–	2.12 ± 0.00	4.96 ± 0.00
α-Humulene	20.48	1442	1452	5.49 ± 0.40	8.04 ± 0.22	5.30 ± 0.00	9.07 ± 0.03
*allo*-aromadendrene	20.68	1448	1458	0.01 ± 0.00	–	0.01 ± 0.00	0.18 ± 0.01
(*E*)-β-farnesene	20.88	1455	1454	0.41 ± 0.04	0.33 ± 0.02	0.41 ± 0.01	0.92 ± 0.02
Selina-4,11-diene	21.22	1466	1476	0.06 ± 0.01	0.06 ± 0.00	0.06 ± 0.01	0.09 ± 0.01
γ-Muurolene	21.27	1467	1478	0.08 ± 0.02	0.08 ± 0.01	0.03 ± 0.01	0.08 ± 0.00
β-Selinene	21.46	1474	1489	0.50 ± 0.01	0.73 ± 0.03	0.74 ± 0.05	1.1 ± 0.01
9-*epi*-(*E*)-caryophyllene	21.56	1476	1475	–	–	–	0.13 ± 0.01
α-Selinene	21.73	1482	1498	1.41 ± 0.11	2.04 ± 0.07	1.39 ± 0.03	1.00 ± 0.01
α-Zingiberene	22.07	1493	1493	–	–	–	0.12 ± 0.00
α-Bulnesene	22.14	1494	1509	2.26 ± 0.45	2.25 ± 0.07	3.37 ± 0.02	5.95 ± 0.03
γ-Cadinene	22.35	1503	1513	1.61 ± 0.10	1.98 ± 0.00	1.57 ± 0.00	3.00 ± 0.00
β-Bisabolene	22.36	1503	1505	1.83 ± 0.14	2.18 ± 0.07	1.74 ± 0.03	3.2 ± 0.02
(*E*,*E*)-α-farnesene	22.45	1505	1505	–	–	–	2.14 ± 0.02
β-Himachalene	22.47	1506	1512	1.84 ± 0.17	1.97 ± 0.04	1.43 ± 0.04	–
δ-Cadinene	22.71	1514	1522	0.11 ± 0.01	0.10 ± 0.00	0.07 ± 0.00	0.09 ± 0.00
β-Sesquiphellandrene	22.77	1516	1521	0.10 ± 0.00	0.12 ± 0.01	0.11 ± 0.01	0.19 ± 0.00
Selina-4(15),7(11)-diene	22.93	1522	1544	5.96 ± 0.56	5.36 ± 0.10	3.39 ± 0.07	2.66 ± 0.03
Selina-3,7(11)-diene	23.12	1528	1545	9.60 ± 0.81	9.22 ± 0.12	5.61 ± 0.09	3.94 ± 0.05
(E)-α-bisabolene	23.37	1537	1540	2.12 ± 0.21	2.61 ± 0.07	2.75 ± 0.04	6.54 ± 0.07
Germacrene B	23.53	1542	1559	1.62 ± 0.36	1.94 ± 0.09	0.01 ± 0.00	0.77 ± 0.02
(*E*)-nerolidol	24.02	1557	1561	0.01 ± 0.01	0.02 ± 0.00	0.72 ± 0.06	0.27 ± 0.02
Caryophyllene oxide	24.26	1569	1583	0.52 ± 0.01	0.79 ± 0.08	0.29 ± 0.03	5.09 ± 0.37
Humulene epoxide I	24.75	1585	1598	–	–	–	0.03 ± 0.00
Guaiol	24.83	1587	1600	2.59 ± 0.03	2.02 ± 0.11	6.99 ± 0.30	0.02 ± 0.00
Humulene epoxide II	25.00	1593	1608	0.29 ± 0.02	0.38 ± 0.04	0.58 ± 0.00	1.08 ± 0.09
5-*epi*-7-*epi*-α-eudesmol	25.01	1594	1607	–	–	0.44 ± 0.00	–
10-*epi*-γ-eudesmol	25.31	1604	1622	2.91 ± 0.06	2.48 ± 0.13	6.88 ± 0.31	0.06 ± 0.00
γ-Eudesmol	25.70	1618	1630	0.14 ± 0.02	0.12 ± 0.01	0.75 ± 0.01	–
Caryophylla-4(12),8(13)-dien-5β-ol	25.81	1622	1639	–	–	–	0.04 ± 0.01
β-Eudesmol	26.16	1635	1649	0.67 ± 0.02	0.52 ± 0.04	2.11 ± 0.17	–
α-Eudesmol	26.26	1639	1652	0.94 ± 0.05	0.71 ± 0.06	2.97 ± 0.20	–
Bulnesol	26.70	1655	1670	1.61 ± 0.03	1.19 ± 0.08	4.27 ± 0.32	–
14-Hydroxy-(*Z*)-caryophyllene	26.82	1661	1666	–	–	–	0.02 ± 0.00
*epi*-α-bisabolol	27.25	1675	1683	1.80 ± 0.23	2.44 ± 0.30	3.62 ± 0.47	6.00 ± 0.91
α-Bisabolol	27.33	1677	1685	0.22 ± 0.03	0.22 ± 0.02	0.23 ± 0.01	0.13 ± 0.05
Eudesm-7(11)-en-4-ol	27.40	1680	1700	0.20 ± 0.01	0.23 ± 0.01	0.18 ± 0.02	–
Cannabidivarin	39.94	2203		–	-	0.02 ± 0.01	–
Cannabicitran	41.01	2255		–	–	0.02 ± 0.01	–
Cannabidiol	44.16	2408		0.15 ± 0.00	0.14 ± 0.00	5.33 ± 1.39	0.06 ± 0.00
Cannabichromene	44.44	2430		0.06 ± 0.00	0.04 ± 0.00	–	–

^a^Temperature-programmed linear retention index experimentally determined by comparison with a mixture of C*8*-C*30 n*-alkanes.

^b^RI value taken from Adams (2007).

The EO chemical profile of the four strains was also different. Cherry Wine and Red Bordeaux had higher concentrations of myrcene compared with Umpqua and T&H. Limonene was around 4–5% in Cherry Wine, Red Bordeaux and Umpqua but < 1% in T&H. Conversely, (*E*)-caryophyllene was much higher in T&H (30.1%) and lower in the other 3 hemp strains. α-*trans*-Bergamotene was also higher in T&H and much lower in the other 3 hemp strains.

α-Humulene and α-bulnesene, (*E*)-α-bisabolene, caryophyllene oxide, and *epi*-α-bisabolol were also higher in the EO of T&H and lower in the EO of the other three strains. The highest concentration of guaiol, 10-*epi*-γ-eudesmol, bulnesol, and cannabidiol (5.3%) were found in the EO of Umpqua. The concentration of cannabidiol was < 0.2% in the EO of the other three strains. α-Guaiene was only found in T&H and in Umpqua, cannabidivarin and cannabicitran were only detected in the EO of Umpqua, (*E*,*E*)-α-farnesene (2.1%) was only found in the EO of T&H.

Cherry Wine EO contained myrcene (23.2%), (*E*)-caryophyllene (16.7%), selina-3,7(11)-diene (9.6%), as the three main constituents (> 10% of total oil) (Table 1). The Red Bordeaux main EO constituents were (*E*)-caryophyllene (~ 20%), myrcene (16.6%), selina-3,7(11)-diene (9.6%), and α-humulene (8.0%).

The EO of Umpqua had (*E*)-caryophyllene (18.2%) as the main constituent, other constituents included guaiol (7.0%), 10-*epi*-γ-eudesmol (6.9%), selina-3,7(11)-diene (5.6%), cannabidiol (5.3%), and α-humulene (5.3%). (*E*)-Caryophyllene (30.5%) was the main constituent of T&H strain; other constituents included α-humulene (9.1%), (*E*)-α-bisabolene (6.5%), *epi*-α-bisabolol (6.0%), α-bulnesene (6.0%), and caryophyllene oxide (5.1%) (Table 1).

### Effect of distillation on cannabinoids

The distillation of hemp biomass resulted in two high-value products: essential oil (EO) and distilled biomass with largely preserved but altered cannabinoids because of the decarboxylation that occurs during the distillation. Most notable, the distillation of hemp resulted in apparent decarboxylation and conversion of cannabinoids in the distilled biomass. One of the notable conversions of interest is the decarboxylation of CBD-A into CBD (Table 2). This was observed in all four different strains (chemovars). Distillation of the biomass slightly increased the concentration of total CBD in Cherry Wine and decreased it slightly in Red Bordeaux. Overall, the total CBD ranged from 2.3 to 11.7% and from 2.1 to 12.7% in the non-distilled and distilled biomass, respectively.

**Table 2:** Cannabinoid content (%) in distilled and not distilled biomass of 4 varieties, transplanted autoflower type hemp plants (mean ± std.err.; n = 2).

Cannabinoids	‘Cherry Wine’		‘Red Bordeaux’		‘Umpqua’		Chopped biomass (T & H)	
	Not distilled	Distilled 240 min	Not distilled	Distilled 240 min	Not distilled	Distilled 240 min	Not distilled	Distilled 240 min
	%							
CBC	0.16 ± 0.01	0.67 ± 0.01	0.21 ± 0.00	0.59 ± 0.01	0.09 ± 0.00	0.46 ± 0.01	0.00 ± 0.00	0.12 ± 0.01
CBC-A	0.56 ± 0.01	0.03 ± 0.00	0.53 ± 0.00	0.00 ± 0.00	0.46 ± 0.01	0.04 ± 0.00	0.11 ± 0.01	0.00 ± 0.00
CBD	2.20 ± 0.09	12.30 ± 0.00	2.87 ± 0.13	10.10 ± 0.00	1.26 ± 0.01	11.35 ± 0.15	0.33 ± 0.03	2.07 ± 0.10
CBD-A	10.06 ± 0.25	0.42 ± 0.01	9.89 ± 0.45	0.32 ± 0.01	11.90 ± 0.20	0.74 ± 0.00	2.29 ± 0.16	0.04 ± 0.00
CBD-Total	11.00 ± 0.30	12.70 ± 0.00	11.5 ± 0.50	10.40 ± 0.00	11.70 ± 0.20	12.05 ± 0.15	2.33 ± 0.16	2.11 ± 0.10
CBDV	0.02 ± 0.02	0.25 ± 0.01	0.04 ± 0.00	0.23 ± 0.00	0.00 ± 0.00	0.13 ± 0.01	0.00 ± 0.00	0.00 ± 0.00
CBDV-A	0.08 ± 0.02	0.00 ± 0.00	0.13 ± 0.01	0.00 ± 0.00	0.12 ± 0.00	0.00 ± 0.00	0.00 ± 0.00	0.00 ± 0.00
CBG	0.10 ± 0.01	0.19 ± 0.01	0.10 ± 0.00	0.14 ± 0.00	0.11 ± 0.01	0.32 ± 0.06	0.00 ± 0.00	0.00 ± 0.00
CBG-A	0.27 ± 0.01	0.00 ± 0.00	0.15 ± 0.02	0.00 ± 0.00	0.25 ± 0.00	0.00 ± 0.00	0.00 ± 0.00	0.00 ± 0.00
CBN	0.00 ± 0.00	0.04 ± 0.01	0.00 ± 0.00	0.04 ± 0.00	0.00 ± 0.00	0.07 ± 0.00	0.00 ± 0.00	0.00 ± 0.00
δ-9 THC	0.18 ± 0.01	0.35 ± 0.01	0.22 ± 0.01	0.27 ± 0.00	0.154 ± 0.01	0.36 ± 0.02	0.00 ± 0.00	0.05 ± 0.00
THC-A	0.21 ± 0.00	0.00 ± 0.00	0.18 ± 0.00	0.00 ± 0.00	0.34 ± 0.02	0.00 ± 0.00	0.04 ± 0.01	0.00 ± 0.00
THC-total	0.36 ± 0.01	0.35 ± 0.01	0.38 ± 0.00	0.20 ± 0.00	0.45 ± 0.03	0.36 ± 0.02	0.00 ± 0.00	0.00 ± 0.00
Cannabinoids-total	13.85 ± 0.35	14.25 ± 0.05	14.35 ± 0.55	11.7 ± 0.00	14.65 ± 0.25	13.50 ± 0.20	2.77 ± 0.20	2.28 ± 0.11

Similarly, distillation resulted in the decarboxylation of CBC-A into CBC; the concentration of CBC in the distilled biomass increased 4.1, 2.8, and 5.2 times in Cherry Wine, Red Bordeaux, Umpqua relative to the non-distilled biomass, respectively, and from 0 to 0.123%, in T&H. There was concomitant decrease of CBC-A from non-distilled to distilled biomass.

Similar tendency was observed with the conversion of CBG-A into CBG in Cherry Wine, Red Bordeaux, and Umpqua; CBG-A in the distilled biomass was below the detection limit of the instrument. Overall, distillation resulted in slight decrease of total CBG in Cherry Wine and Red Bordeaux and slight increase in the total CBG in Umpqua. The CBG-A and CBG in T&H were both under the detection limit.

The concentration of CBN in not-distilled biomass was under the detection limit and was 0.041, 0.035, and 0.075% in the distilled biomass of Cherry Wine, Red Bordeaux and Umpqua, respectively, while it was under the detection limit in T&H.

As expected, distillation resulted in conversion of all THC-A into THC. This has both practical and legal importance; some states limit the concentration of THC in hemp while others limit the concentration of total THC. The concentration of THC in the distilled biomass was 197, 124, and 236% in Cherry Wine, Red Brodeau, and Umpua, relative to their respective concentrations in the not-distilled biomass, respectively. Overall, distillation tended to increase the concentration of total THC in Cherry Wine but decreased it a bit in the rest of the hemp strains (Table 2).

### Scanning electron microscopy (SEM) of the distilled biomass

**Figure 3:**
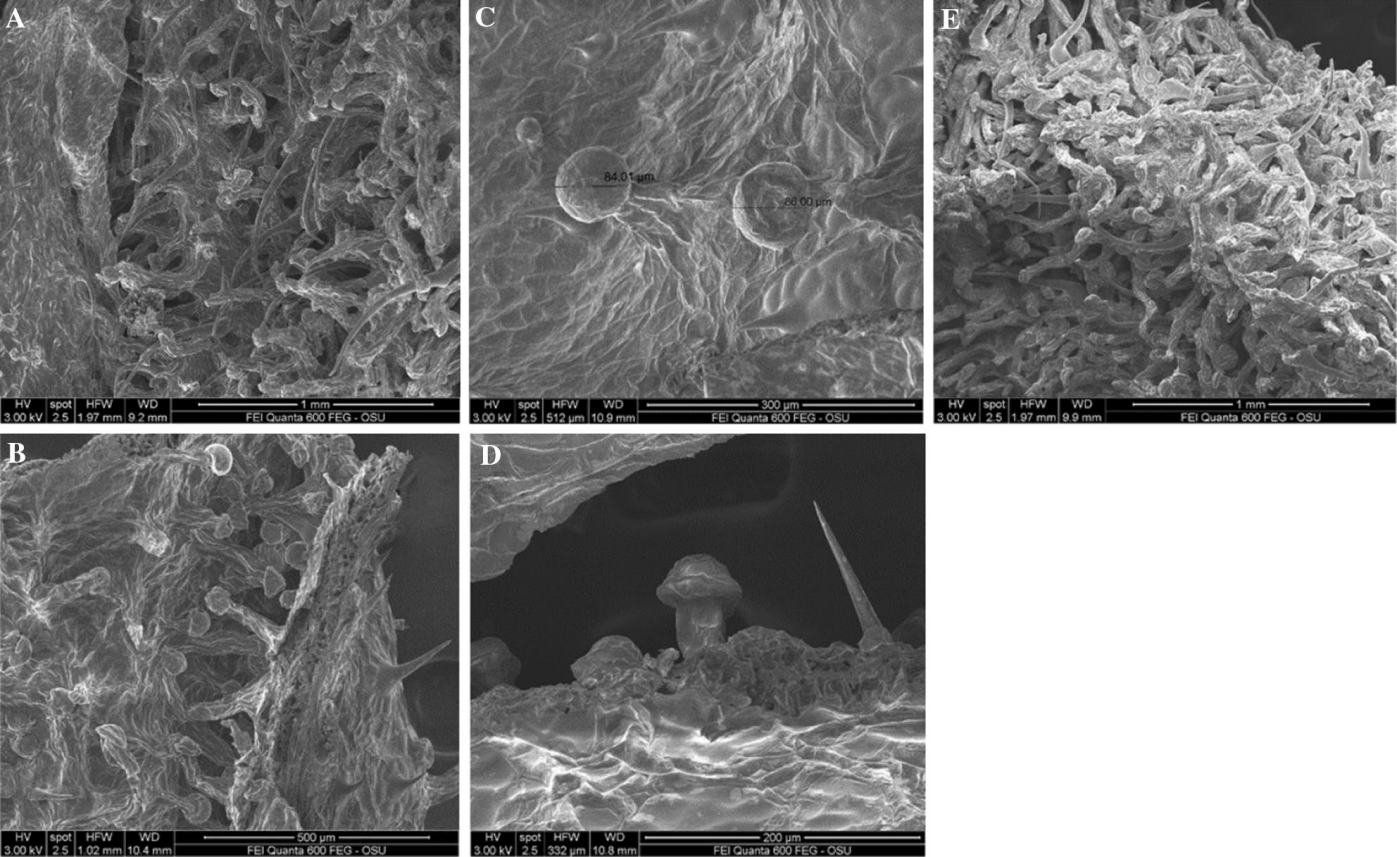
(**A**) Red Bordeaux extracted flower/leaf parts. (**B**) Red Bordeaux extracted flower/leaf parts. (**C**) Red Bordeaux extracted leaf with non-destructed glandular trichomes. (**D**) Red Bordeaux extracted leaf with non-destructed glandular trichomes and well preserved cystolithic trichomes. (**E**) Cherry Wine extracted flower/leaf parts.

**Figure 4:**
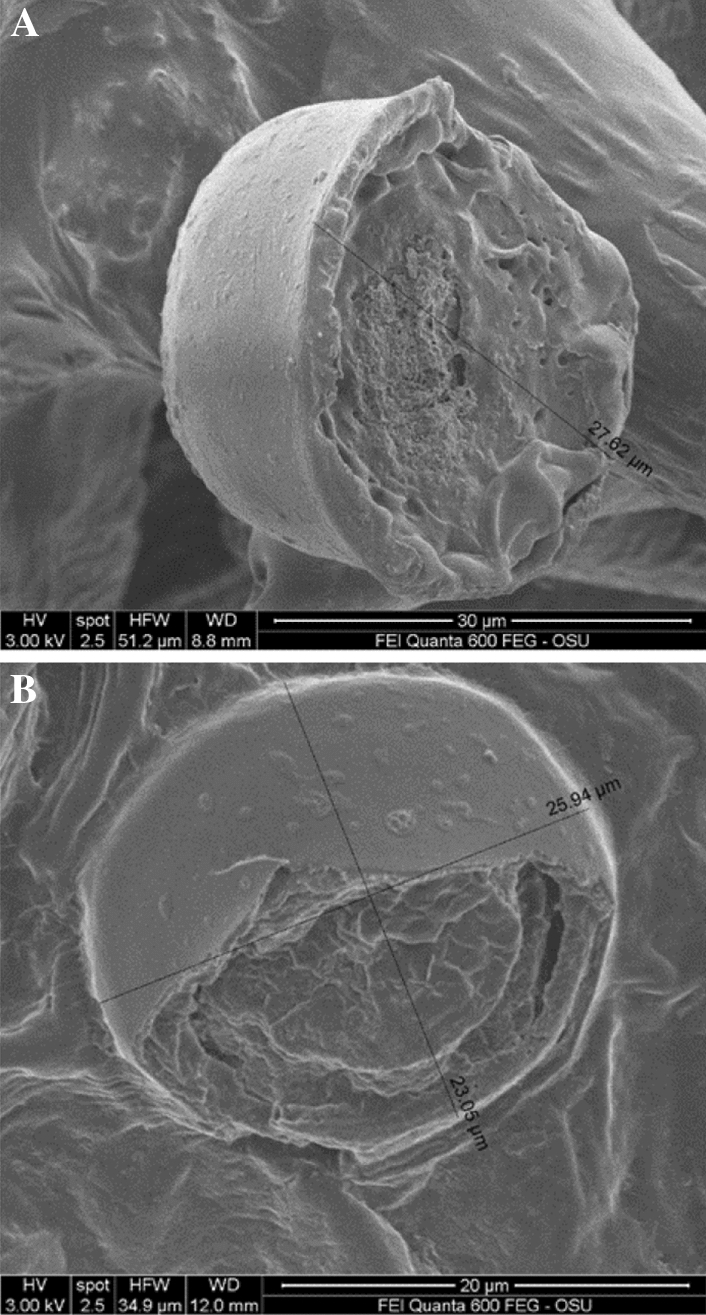
(**A**) T&H non-extracted leaf with part of the sessile gland missing probably due to the mechanical chopping of the biomass, revealing resinoid substance inside that could be a mix of the cannabinoids and some of the terpenes that did not volatilize. (**B**) T&H Extracted leaf part with part of the sessile gland missing revealing resinoid substance inside that could be the cannabinoids and some of the non-extracted terpenes.

Scanning electron microscopy (SEM) analyses revealed that most of the glandular trichomes in the distilled biomass were not disturbed, they were not open (Fig. 3A–E). That suggest a possibility for terpenes evaporation through the epidermal membrane covering the glandular trichomes leaving the cannabinoids in the trichomes. This explained the fact that distillation resulted in terpene extraction while the cannabinoids remained in the distilled material. Furthermore, mechanical harvest and chopping of the T&H biomass resulted in damage of some of the glandular trichomes (Fig. 4A), however, it seems while some of the terpenes may have evaporated, some may have formed a resinoid-like slush with the cannabinoids that did not volatilize. Furthermore, an open sessile gland in T&H after the extraction of the EO (Fig. 4B) indicates similar resinoid-like substance that can be assumed to contain mostly cannabinoids.

## Discussion

This study demonstrated that distillation of hemp biomass may extract the terpenes (EO) and leave the cannabinoids in the distilled biomass that can be further extracted. This presents an opportunity for valorization of hemp biomass because of the resulting two high-value products: essential oil (EO) and distilled biomass with largely preserved but altered (into desirable chemical forms) cannabinoids because of the decarboxylation that occurs during the distillation.

Secondly, the study reveal that the above effects may depend on the specific variety (strain, cultivar) as some CBD was transferred into the EO of one of the tested strains but not in the other three. Still, most of the CBD stayed in the distilled biomass. The extracted biomass did not possess any aroma because the volatile terpenes were extracted. That presents an opportunity for the extracted biomass to be included in various products with targeted designed aroma and flavor of choice.

The SEM analyses of distilled biomass revealed that the thin layer covering the glands of the glandular trichomes were not open suggesting that terpenes may have moved through this membrane during distillation leaving the cannabinoids in the glands.

Third, the EO yield, and profile of different strains can differ significantly as a function of the variety (genetics); the major EO constituents can be either the same but in the different concentration gradients, or the 3–5 main EO constituents could be different in different strains. That presents an opportunity to obtain EO with specific composition and subsequently aroma, that would be of interest to the aroma and flavor industries.

Overall, the EO yield in this study clearly showed that the hemp strains tested in this study were very different from the typical registered industrial hemp varieties listed in the European Union (EU)^22^ and in Canada^23^. The EO yield of the hemp strains in this study varied from 0.72 to 1.85% in dried flowers and upper leaves except for the chopped whole plant biomass of T&H which was 0.37%. Recent literature data showed that the EO yield of 8 industrial hemp breeding lines was between 0.06 and 0.14%, while the EO yield of other 8 registered industrial hemp varieties was 0.1–0.2% (mL per 100 g air-dried hemp biomass)^24^. Other studies on industrial hemp have reported EO yield of 0.04–0.3%^3,5,6,9,25–27^.

There are two reasons for the higher EO content of the high-value (high-cannabinoids) hemp used in this study: (1) the four strains in this study were selected in the past from the medical or illicit marijuana strains that have different architecture (phenotype) and genotype than the registered industrial hemp varieties; and (2) three of the strains in this study were established using feminized seed and care was taken to avoid pollination and fertilization of the female flowers, that results in higher density of glandular trichomes (Fig. 1D). The T&H was grown until late, and harvested with a forage chopper that resulted in EO losses (Fig. 4A,B).

Myrcene and (*E*)-caryophyllene were two of the main EO constituents in the hemp strains in this study. Myrcene has been reported as a major EO constituent in industrial hemp, ranging from negligible amounts to 25% of the EO^3,5,21,26–29^. Also, myrcene is found in higher concentrations in hops EO depending on the distillation time^30^. The importance and the use of myrcene, acyclic monoterpene, has been reviewed^31^; it is a constituent in the EO of many other species such as hop, lemongrass, nutmeg, sage, rosemary and others^31,32^. However, the major raw material for myrcene has been turpentine^31^. Other chemicals such as menthol, geraniol, nerol, linalool can be commercially produced from myrcene, and these products have wide and various applications such as flavor and fragrance agents, in insect repellents, vitamins and also in polymers, pharmaceuticals and surfactants^31^. However, myrcene has been touted as potential carcinogen, and suggested that food and beverages with myrcene should be monitored^32^. Indeed, research has shown myrcene was linked to tumor in the urinary tracts of rodents although no data is available for humans^33^.

(*E*)-Caryophyllene, a bicyclic sesquiterpene, has been reported as a constituent of industrial hemp EO ranging from 14 to 33% of the total oil^3,26,28^. (*E*)-Caryophyllene is a known anti-inflammatory agent, that possesses also analgesic action; it is used as food additive/flavoring agent, has many other biological properties^34,35^. It is found in industrial hemp varieties from 22 to 55% in registered varieties and from 11 to 22% of the EO of breeding lines^36^. (*E*)-Caryophyllene is considered a dietary cannabinoid and in vivo, it was reported to act as non-psychotropic CB2 receptor ligand in foodstuff^37^. (*E*)-Caryophyllene is found in the EO of other plant species such as peppermint (*Mentha* × *piperita* L.), common basil (*Ocimum basilicum* L.), oregano (*Origanum vulgare* L.) black pepper (*Piper nigrum* L.), and has been known to possess insecticidal, acaricidal, repellent, and antifungal properties^10,35,38^.

Recent study on 8 registered industrial hemp varieties in Europe (in Serbia, which is approximately at the same latitude as Oregon) has shown the following main EO constituents: (*E*)-caryophyllene 11–22% and 15.4–29.6%; α-humulene 4.4–7.6% and 5.3–11.9%; caryophyllene oxide 8.6–13.7%^36^. The major EO constituents of the U.S. high-cannabinoid hemp strain that was grown in the close vicinity to the above study in Serbia had different chemical profile, with major constituents as myrcene (9.2 to 12%), (*E*)-caryophyllene (6.5 to 7.5%), limonene (3.8 to 4.2%), (*E*)-β-ocimene (5.3 to 5.6%) and α-bisabolol (3.9 to 4.4%)^36^. Therefore, we may postulate that the high-cannabinoid U.S. hemp strains will synthesize and accumulate similar cannabinoids and EO amount and composition in other remote geographic areas at similar latitude.

## Conclusions

This study elucidated the effect of the steam distillation of four high-cannabinoids hemp strains on changes in the content and compositional profile of cannabinoids. The study demonstrated a simple method for valorization of CBD-hemp through the production of two high-value chemicals; EO and cannabinoids with improved profile through the conversion of CBD-A into CBD, CBC-A into CBC, CBDV-A into CBDV, CBG-A into CBG, and THC-A into THC. In addition, the distilled biomass contained CBN while the non-distilled did not. Distillation improved cannabinoids profile; e.g. the distilled hemp biomass had 3.4 times higher CBD in variety Red Bordeaux, 5.6 times in Cherry Wine, 9 times in variety Umpqua, and 6 times in T&H compared to the original non-distilled samples, respectively. The main 3 EO constituents were similar but in different ratio. The distillation converted most of the THC-A into THC reducing total THC in the process, which carries practical and legal importance because of the rapidly changing legal environment in the U.S. and across the world. Scanning electron microscopy (SEM) analyses revealed that most of the glandular trichomes in the distilled biomass were not disturbed (open); that suggest a possibility for terpenes evaporation through the epidermal membrane covering the glandular trichomes leaving the cannabinoids in the trichomes.

## Methods

### Plant material

The plant material utilized in this study was from varieties (strains) of cultivated hemp (*Cannabis sativa* L.) in the United States and this is not an endangered species at risk of extinction. The collection of plant tissue research specimens was acquired (including transportation) conformed scrupulously to procedures and regulations adopted under international legal agreements. In addition, the plant material sampling, transportation, and handling was in compliance with the U.S. federal and Oregon state legislations. Certified and compliant (THC < 0.3% in dry biomass) organically grown CBD-hemp strains (also called chemovars, varieties) Red Bordeaux, Cherry Wine and Umpqua (flowers and some upper leaves) and a T&H strain that included chopped whole-plant biomass were donated by two licensed Oregon hemp producers. The original Certificates of analyses are kept and available from the authors. We are using “strain” to denote non-registered hemp variety (cultivar); this is a common term in the hemp industry in the U.S.

### Distillation of the essential oil (EO)

Representative subsamples in 3 replicates from each of the four hemp strains were subjected to steam distillation for 240 min in 2-L steam distillation apparatuses as described previously^39^. The first drop of the EO in the separator part of the apparatus was considered the beginning of the distillation. After 240 min non-stop distillation, the power was switched off, the heat source was removed, the EOs were collected in glass vials and stored in a freezer. Later, the EO was separated from the remaining water in the vials, its weight was taken on analytical scale, and transferred to a freezer again until the gas chromatography (GC) analyses could be performed.

The remaining hemp biomass was removed from the bioflask and spread for drying at T around 30 °C at forced air. After the biomass reached a constant weight, subsamples were generated for cannabinoid extraction.

### Cannabinoid extraction and identification

Subsamples from non-extracted (original) and extracted biomass was submitted for cannabinoid analyses and characterization to the Columbia Laboratories in Portland, OR (https://www.columbialaboratories.com/), a commercial laboratory that is ISO 17025:2017 accredited, as well as TNI certified. The method of cannabinoid extraction and analyses was JAOAC 2015 V98-6^20^ and the instrumentation was HPLC–DAD Agilent 1200 series (Agilent Technologies, Inc. Santa Clara, CA, U.S.A).

### Gas chromatography-mass spectrometry (GC–MS) analyses of the essential oils

A gas chromatograph Agilent 6890 N equipped with a single quadrupole mass spectrometer 5973 N was used. The stationary phase was a HP-5MS (30 m l. × 0.25 mm i.d., 0.1 mm f.t., Folsom, CA, USA) made up of 5% phenylmethylpolysiloxane; the mobile phase was helium (99.999%) flowing at 1 mL/min. The temperature of the oven was programmed as follows: 60 °C held for 5 min, then increase up to 220 °C at 4 °C/min, finally 11 °C/min up to 280 °C held for 15 min. Once diluted in n-hexane (dilution ratio 1:100) the hemp EO samples were injected (2 μL) through an auto-sampler 7863 (Agilent, Wilmingotn, DE) in the inlet of GC taken at 280 °C using the split mode (split ratio 1:50). Peaks were acquired in full scan mode (29–400 m*/z*) using the electron impact (EI) mode at 70 eV. Chromatograms were analyzed by the Enhanced Data Analysis program of Agilent G1701DA GC/MSD ChemStation. In addition, the NIST Mass Spectral Search Program for the NIST/EPA/NIH EI was used for peak assignment. Mass spectra (MS) of peaks were compared with those stored in ADAMS^40^ (Adams, 2007), NIST 17 and FFNSC3 libraries. The temperature-programmed retention indices (RI) were determined using a homologue mixture of C8-C30 n-alkanes (Merk, Milan, Italy) and computed by the following formula (ref.^41^):

$$RIx=100n+\frac{100\left(tx-tn\right)}{tn+1-tn},$$

where n is the number of carbon atom of the alkane eluting before the unknown peak, tx the retention time of the unknown peak, tn the retention time of the alkane eluting before the unknown peak and tn + 1 the retention time of the alkane eluting after the unknown peak. The combination of the MS overlapping and RI coherence with respect to those reported in the aforementioned libraries was used to assign the peak. Furthermore, for the following compounds the identity was confirmed by comparison with analytical standard: α-pinene, camphene, sabinene, β-pinene, myrcene, *p*-cymene, limonene, 1,8-cineole, (*Z*)-β-ocimene, (*E*)-β-ocimene, γ-terpinene, terpinolene, linalool, borneol, α-terpineol, (*E*)-caryophyllene, α-humulene, (*E*)-β-farnesene, (*E*)-nerolidol, caryophyllene oxide, cannabidiol (Merck). The relative peak area percentages were obtained from the chromatograms without using correction factors. The GC–MS response resulted similar to that of GC-FID as determined previously^21^.

### Scanning electron microscopy (SEM) analysis of hemp flowers, glands, leaves and stems

The scanning electron microscope (SEM) used in this investigation of hemp biomass extracted and non-extracted samples was an FEI Quanta 600 SEM (ThermoFisher Scientific/FEI, Hillsboro, OR, U.S.A.) at the Microscopy Facility at Oregon State University, (https://emfacility.science.oregonstate.edu/). Samples were placed into a fixative, 1% paraformaldehyde and 2.5% glutaraldehyde in 0.1 M sodium cacodylate buffer with pH 7.4, soaked in the fixative for 2 h, rinsed in 0.1 M cacodylate buffer, 15 min each, and dehydrated in acetone (10%, 30%, 50%, 70%, 90%, 95%, 100%), 10–15 min each, followed by critical point drying (two ‘bomb flushes’ at chamber pressure to 5 °C, fill chamber with CO_2_). The samples were left to vent for 5 min, and then, the procedure was repeated. The dry samples were mounted onto an aluminum SEM stub with double stick carbon tape. Samples were sputter coated with a Cressington (Cressington Scientific Instruments, Watford, U.K.) 108A sputter coater from Ted Pella with Au/Pd, 60/40 mix.

## References

[1] Allen C, Whitney B (2019). The Field of Dreams. An Economic Survey of the United States Hemp Cultivation Industry.

[2] Andre CM, Hausman JF, Guerriero G (2016). *Cannabis sativa*: The plant of the thousand and one molecules. Front. Plant Sci..

[3] Booth JK, Page JE, Bohlmann J (2017). Terpene synthases from *Cannabis sativa*. PLoS ONE.

[4] Flores-Sanchez IJ, Verpoorte R (2008). Secondarymetabolismin Cannabis. Phytochem. Rev..

[5] Bedini S, Flamini G, Cosci F, Ascrizzi R, Benelli G, Conti B (2016). *Cannabis sativa* and *Humulus lupulus* essential oils as novel control tools against the invasive mosquito *Aedes albopictus* and fresh water snail *Physella acuta*. Ind. Crop Prod..

[6] Benelli G, Pavela R, Petrelli R, Cappellacci L, Santini G, Fiorini D, Sut S, Dall'Acqua S, Canale A, Maggi F (2018). The essential oil from industrial hemp (*Cannabis sativa* L.) by-products as an effective tool for insect pest management in organic crops. Ind. Crop Prod..

[7] Nadal X, Río C, Del Casano S, Palomares B, Ferreiro-Vera C, Navarrete C, Sánchez-Carnerero C, Cantarero I, Bellido ML, Meyer S, Morello G, Appendino G, Muñoz E (2017). Tetrahydrocannabinolic acid is a potent PPARγ agonist with neuroprotective activity. Br. J. Pharmac..

[8] Nafis A, Kasrati A, Jamali CA, Mezrioui N, Setzer W, Abbad A, Hassani L (2019). Antioxidant activity and evidence for synergism of *Cannabis sativa* (L.) essential oil with antimicrobial standards. Ind. Crop Prod..

[9] Zengin G, Menghini L, Sotto A, Di Mancinelli R, Sisto F, Carradori S, Cesa S, Fraschetti C, Filippi A, Angiolella L, Locatelli M, Mannina L, Ingallina C, Puca V, D'Antonio M, Grande R (2018). Chromatographic analyses, in vitro biological activities, and cytotoxicity of *Cannabis sativa* L. essential oil: A multidisciplinary study. Molecules.

[10] Tabari MA, Khodashenas A, Jafari M, Petrelli R, Cappellacci L, Nabissi M (2020). Acaricidal properties of hemp (*Cannabis sativa* L.) essential oil against *Dermanyssus gallinae* and *Hyalomma dromedarii*. Ind. Crop Prod..

[11] Small E, Cronquist A (1976). A practical and natural taxonomy for cannabis. Taxon.

[12] Small E (2015). Evolution and classification of *Cannabis sativa* (marijuana, hemp) in relation to human utilization. Bot. Rev..

[13] Raman V, Lata H, Chandra S, Khan IA, ElSohly MA, Chandra S, Lata H, ElSohly MA (2017). Morpho-anatomy of marijuana (*Cannabis sativa* L.). *Cannabis sativa* L.-Botany and Biotechnology.

[14] Small E (2017). Cannabis Guide.

[15] Small E, Naraine SGU (2016). Expansion of female sex organs in response to prolonged virginity in Cannabis sativa (marijuana). Genet. Resour. Crop Evol..

[16] Zirpel B, Stehle F, Kayser O (2015). Production of Δ9-tetrahydrocannabinolic acid from cannabigerolic acid by whole cells of Pichia (Komagataella) pastoris expressing Δ9-tetrahydrocannabinolic acid synthase from *Cannabis sativa* L.. Biotechnol. Lett..

[17] ElSohly MA, Slade D (2005). Chemical constituents of marijuana: The complex mixture of natural cannabinoids. Life Sci..

[18] Russo E (2011). Taming THC: Potential cannabis synergy and phytocannabinoid-terpenoid entourage effects. Br. J. Pharmac..

[19] Brenneisen R, ElSohly M (2007). Chemistry and analysis of phytocannabinoids and other cannabis constituents”. Marijuana and the Cannabinoids Forensic Science and Medicine.

[20] Giese MW, Lewis MA, Giese L, Smith KM (2015). Development and validation of a reliable and robust method for the analysis of cannabinoids and terpenes in Cannabis. J. AOAC Int..

[21] Fiorini D, Scortichini S, Bonacucina G, Greco NG, Mazzara E, Petrelli R (2020). Cannabidiol-enriched hemp essential oil obtained by an optimized microwave-assisted extraction using a central composite design. Ind. Crop Prod..

[22] European Commission. *EU Plant Variety Database. Agricultural Species. A-85-Hemp Cannabis sativa* (2020). https://ec.europa.eu/food/plant/plant_propagation_material/plant_variety_catalogues_databases/search/public/index.cfm?event=SearchVariety&ctl_type=A&species_id=240&variety_name=&listed_in=0&show_current=on&show_deleted (Accessed 4 March 2021).

[23] Government of Canada. *List of Approved Cultivars for the 2020 Growing Season: Industrial Hemp Varieties Approved for Commercial Production* (2021). https://www.canada.ca/en/health-canada/services/drugs-medication/cannabis/producing-selling-hemp/commercial-licence/list-approved-cultivars-cannabis-sativa.html (Accessed 4 March 2021).

[24] Zheljazkov VD, Sikora V, Semerdjieva IB, Kačániová M, Astatkie T, Dincheva I (2020). Grinding and fractionation during distillation alter hemp essential oil profile and its antimicrobial activity. Molecules.

[25] Bertoli A, Tozzi S, Pistelli L, Angelini LG (2010). Fiber hemp inflorescences; from crop-residues to essential oil production. Ind. Crop Prod..

[26] Benelli G, Pavela R, Lupidi G, Nabissi M, Petrelli R, Ngahang Kamte S, Cappellacci L, Dennis Fiorini D (2018). The crop-residue of fiber hemp cv. Futura 75: From a waste product to a source of botanical insecticides. Environ. Sci. Pollut. Res..

[27] Nissen L, Zatta A, Stefanini I, Grandi S, Sgorbati B, Biavati B, Monti A (2010). Characterization and antimicrobial activity of essential oils of industrial hemp varieties (*Cannabis sativa* L.). Fitoterapia.

[28] Fiorini D, Molle A, Nabissi M, Santini G, Benelli G, Maggi F (2019). Valorizing industrial hemp (*Cannabis sativa* L.) by-products: Cannabidiol enrichment in the inflorescence essential oil optimizing sample pre-treatment prior to distillation. Ind. Crop Prod..

[29] Nagy DU, Cianfaglione K, Maggi F, Sut S, Dall'Acqua S (2019). Chemical characterization of leaves, male and female flowers from spontaneous Cannabis (*Cannabis sativa* L.) growing in Hungary. Chem. Biodivers..

[30] Jeliazkova EA, Zheljazkov VD, Kačániova M, Astatkie T, Tekwani BL (2018). Sequential elution of essential oil constituents during steam distillation of hops (*Humulus lupulus* L.) and influence on oil yield and antimicrobial activity. J. Oleo Sci..

[31] Behr A, Johnen L (2009). Myrcene as a natural base chemical in sustainable chemistry: A critical review. Chemsuschem.

[32] Okaru AO, Lachenmeier DW (2017). The food and beverage occurrence of furfuryl alcohol and myrcene—Two emerging potential human carcinogens?. Toxics.

[33] IARC (International Agency for Research on Cancer). *Studies of Carcinogenicity in Mice and Rats Treated with β-myrcene by Gavage. Table 3.1* (2019). https://www.ncbi.nlm.nih.gov/books/NBK546955/.

[34] Fidyt K, Fiedorowicz A, Strządała L, Szumny A (2016). β-caryophyllene and β-caryophyllene oxide-natural compounds of anticancer and analgesic properties. Cancer Med..

[35] Francomano F, Caruso A, Barbarossa A, Fazio A, La Torre C, Ceramella J, Mallamaci R, Saturnino C, Iacopetta D, Sinicropi MS (2019). β-caryophyllene: A sesquiterpene with countless biological properties. Appl. Sci..

[36] Zheljazkov VD, Sikora V, Dincheva I, Kačániová M, Astatkie T, Semerdjieva IB, Latkovic D (2020). Industrial, CBD, and wild hemp: How different are their essential oil profile and antimicrobial activity?. Molecules.

[37] Gertsch J, Leonti M, Raduner S, Racz I, Chen JZ, Xie XQ, Altmann KH, Karsak M, Zimmer A (2008). Beta-caryophyllene is a dietary cannabinoid. PNAS.

[38] da Silva RCS, Milet-Pinheiro P, da Silva PCB, da Silva AG, da Silva MV, da Navarro DMF (2015). (E)-Caryophyllene and α-humulene: *Aedes aegypti* oviposition deterrents elucidated by gas chromatography-electrophysiological assay of *Commiphora leptophloeos* leaf oil. PLoS ONE.

[39] Cannon JB, Cantrell CL, Astatkie T, Zheljazkov VD (2013). Modification of yield and composition of essential oils by distillation time. Ind. Crops Prod..

[40] Adams RP (2007). Identification of Essential Oil Components by Gas Chromatography/Mass Spectrometry.

[41] Van den Dool H, Kratz PD (1963). A generalization of the retention index system including linear temperature programmed gas–liquid partition chromatography. J. Chromatogr. A.

